# A Case of Ulcerated Gastric Mass and Anemia due to Strongyloidiasis

**DOI:** 10.1155/2020/8885990

**Published:** 2020-07-20

**Authors:** Monjur Ahmed, Kristen Singer, Cecilia Kelly, Brian O'hara, Curtis Alloy

**Affiliations:** Thomas Jefferson University, Philadelphia, PA, USA

## Abstract

Strongyloidiasis is an intestinal infection caused by the nematode *Strongyloides*, and it is rarely seen in our clinical practice in the United States. Although it remains localized to the gastrointestinal tract most of the time, it can disseminate to other organs when it causes autoinfection in the setting of immunosuppression. The clinical manifestations vary depending on the involved organ. A case of disseminated strongyloidiasis presenting as a case of ulcerated gastric mass and anemia is described here along with a review of the literature on this condition.

## 1. Introduction

*Strongyloides stercoralis* infection is a rare, soil-transmitted, neglected tropical disease. About 50% of patients infected with *Strongyloides* are asymptomatic. As the larvae of *Strongyloides* penetrate the human skin and, then, migrate to the lungs via bloodstream and lymphatics and are, then, swallowed to be transported to the small bowel, patients may present with various cutaneous, pulmonary, and gastrointestinal symptoms during their journey. Patients may notice a mildly itchy skin rash at the site of larval penetration and may develop cough and wheezing during larval migration through the pulmonary parenchyma. But, most commonly, patients generally present with abdominal pain, diarrhea, and occasionally, anorexia, nausea, and vomiting due to congestion, thickening, and ulceration of small intestinal and colonic mucosa. We present here a case of *Strongyloides* infection in an Asian man who not only had abdominal pain but also an ulcerated gastric mass and iron deficiency anemia.

## 2. Key Words

Strongyloidiasis, disseminated strongyloidiasis, gastric mass and strongyloidiasis, anemia and strongyloidiasis, and diagnosis of strongyloidiasis.

## 3. Case

A 68-year-old Lao-speaking male presented with generalized weakness, postprandial dull aching, nonradiating epigastric pain, early satiety, and bloating for about 2 months. He denied any weight loss, fever, or night sweats. He did not have any history of taking nonsteroidal anti-inflammatory drugs, and there was no history of overt gastrointestinal bleeding. He was known to have hyperlipidemia and ascending aortic aneurysm. He was an everyday cigarette-smoker (1/2 pack per day for 25 years) and was a social alcohol drinker. In 1980, he moved from Laos to the United States and worked as a farmer. He had no significant family history of any illness. He was taking aspirin 81 mg daily, atorvastatin 40 mg daily, and multivitamin 1 capsule daily. On examination, he appeared thin built. Vital signs revealed a pulse of 84/minute, blood pressure 121/82 mm Hg, and temperature 98.4°F. Physical examination showed pale conjunctiva, no pedal edema, heart: S1 and S2 regular, no murmur, and breath sounds vesicular, and few crackles at the lung bases. The abdomen was soft and nontender without a palpable mass, and rectal examination was notable for heme-negative stool. Remainder of the examination was unremarkable.

**Figure 1:**
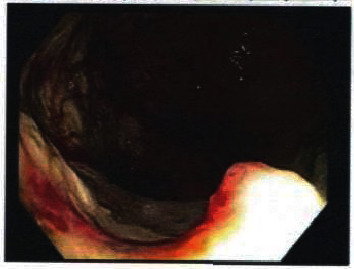
Ulcerated gastric antral mass.

Laboratory studies showed a hemoglobin of 4.7 gm/dl, WBC 10,600/cmm, platelet count 509,000/cmm, MCV 68, serum iron 11 microgram/dl, TIBC 275, iron saturation 4%, and ferritin 10 ng/dl. He was admitted as a case of severe iron deficiency anemia due to chronic occult gastrointestinal blood loss. He was transfused a few units of packed red blood cells with subsequent improvement of his hemoglobin to 8.4 gm/dl. A CT scan showed gastric wall thickening. Upper endoscopy revealed an ulcerated friable mass in the gastric antrum extending proximally to the incisura angularis (Figure 1). Multiple biopsies were taken from the mass, and histopathology showed chronic active gastritis with mucosal necrosis, granulation tissue, and necroinflammatory exudate, negative for *Helicobacter pylori* infection. He was started on ferrous sulfate 325 mg daily and pantoprazole 40 mg daily and was discharged.

**Figure 2:**
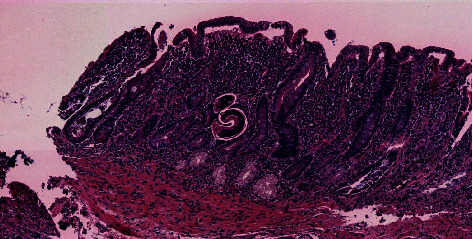
H&E stain of gastric mass showing the nematode and inflammatory cells.

Three months later, the patient was readmitted with epigastric pain, anorexia, nausea, and vomiting. His physical examination was unchanged except for worsened pallor. His hemoglobin was 5.8 gm/dl. He was again transfused 3 units of packed red blood cells, and his hemoglobin increased to 7.9 gm/dl. Upper endoscopy again showed an ulcerated antral mass, with repeat biopsies demonstrating similar histopathology as before. There was no evidence of malignancy. He was discharged home. Two weeks following discharge, he represented to the hospital with generalized weakness and fatigue. His hemoglobin was found to be 4.7 gm/dl, and upper endoscopy demonstrated the same ulcerated mass. Surgery was, then, consulted, and the patient underwent laparoscopic subtotal gastrectomy with Roux-en-Y reconstruction with vagotomy. Histopathology of the ulcerated mass showed ulceration, inflammation, granulation tissue, fibrosis, reactive changes, focal gastritis with intestinal metaplasia, and scattered microscopic foci with morphology consistent with a nematode favoring strongyloides (Figure 2). Postoperatively, stool assay by wet mount after concentration showed strongyloides stercoralis larvae. Serum strongyloides IgG ELISA resulted positive. The patient was discharged home and seen in consultation by infectious diseases, with a plan to start antihelminthic treatment. However, prior to initiating treatment, he developed sepsis and was admitted to an outside hospital, where he unfortunately clinically deteriorated and expired.

## 4. Discussion and Conclusions

Strongyloidiasis is a global infection more commonly seen in Southeast Asia, sub-Saharan Africa, Latin America, Caribbean islands, and Eastern, Southern, and Central Europe [1]. Worldwide, there are 30 to 100 million people infected with *Strongyloides stercoralis* [2]. In the United States, strongyloidiasis is mostly seen in eastern Kentucky and rural Tennessee. But, sporadically, the infection is seen in immigrants from the endemic area, world war II veterans, and residents of mental institutions [3]. *Strongyloides stercoralis* is a potentially lethal cosmopolitan nematode or roundworm also known as human threadworm. It has complex stages of life consisting of a free-living cycle, parasitic cycle, and autoinfection cycle. In the autoinfection cycle, the rhabditiform larvae transform inside the gastrointestinal tract into the infective filariform larvae which can penetrate the intestinal mucosa (internal autoinfection) or perianal skin (external autoinfection) to complete the life cycle. A pathognomonic feature of strongyloides external autoinfection is larva currens in which larvae can be seen creeping under the skin. Once internal autoinfection occurs, the filariform larvae can migrate to the lungs, tracheobronchial tree, and small intestine. If untreated, the autoinfection cycle can result in persistent infection in an individual even after many years of residence in a nonendemic area. Patients with chronic infection may remain asymptomatic for many years except occasional abdominal pain, diarrhea, constipation, and skin rash. Autoinfection may lead to hyperinfection syndrome or disseminated strongyloidiasis if the body's defense mechanism is compromised by different factors, including the use of corticosteroids, immunosuppressive agents or chemotherapy, diabetes mellitus, cirrhosis, malignancy, alcoholism, malnutrition, solid organ transplantation, hematopoietic stem cell transplantation, and HTLV-1 (human T-lymphotropic virus type 1) and HIV (human immunodeficiency virus) infection [4]. In hyperinfection syndrome, the worm burden in the small intestine is excessive due to accelerated normal life cycle of the worm. In disseminated strongyloidiasis, plenty of autoinfective larvae migrate through different organs (the central nervous system, heart, and urinary tract) that are not part of their normal life cycle, and many of them carry enteric organisms from the intestine [5], leading to pneumonia [6], meningitis [7], and sepsis [8]. The case we described had gastric strongyloidiasis as part of his disseminated strongyloidiasis. Cases of disseminated strongyloidiasis with gastroduodenal involvement, including multiple granular lesions, ulcerations, and even duodenal obstruction, have been reported in the literature [9, 10]. However, this is the first case of disseminated strongyloidiasis in which a patient presented with an ulcerated gastric mass and iron deficiency anemia. Intake of aspirin and regular smoking cigarettes could lead to gastric ulcer formation, and the presence of the *Strongyloides* nematode could be a chance finding. But, positive serology, positive stool test, and inflammatory gastric mass with the *Strongyloides* nematode confirmed the diagnosis of disseminated strongyloidiasis. The patient had risk factors for developing strongyloidiasis as he was a native of an endemic area and also worked as a farmer in the United States. But, the obvious reason for developing disseminated strongyloidiasis is unknown. Ivermectin is the treatment of choice for both uncomplicated and complicated strongyloidiasis [11]. Patients should be followed up to confirm eradication, typically with stool testing for the presence of *Strongyloides* larvae 2 to 4 weeks after completing treatment. Patients with persistent infection should receive repeat dosing of ivermectin. Ivermectin was found to be more effective than albendazole or thiabendazole for treatment of strongyloidiasis [11, 12]. In the case of severe disease (hyperinfection syndrome and disseminated strongyloidiasis), ivermectin plus antibiotics against Gram-negative organisms should be initiated and continued for at least 2 weeks. Ideally, treatment should be continued until patients become asymptomatic and daily stool tests for *Strongyloides* larvae remain negative for at least 2 weeks. Successful treatment of disseminated strongyloidiasis with a combination of ivermectin and thiabendazole for 2 weeks has been reported [13]. Suppressive treatment with ivermectin should be given once a month for at least 6 months to patients with persistent immunosuppression who have become asymptomatic after initial treatment [14]. It is also recommended that if patients with hyperinfection/desseminated infection do not respond to initial treatment, they should be tested for underlying immunosuppression such as HIV or HTLV-1 infection. The mortality of patients with hyperinfection syndrome and disseminated strongyloidiasis can be as high as 70 to 90% despite aggressive supportive therapy [15]. In conclusion, strongyloidiasis should be considered in high-risk individuals with inflammatory gastric mass and iron deficiency anemia.
